# Associating learning technology to sustain the environment through green mobile applications

**DOI:** 10.1016/j.heliyon.2019.e01141

**Published:** 2019-01-20

**Authors:** Ramzi A. Haraty, Georges Bitar

**Affiliations:** Department of Computer Science and Mathematics, Lebanese American University, Beirut, Lebanon

**Keywords:** Computer science, Education, Environmental science

## Abstract

Today, there is a great urge to “Go Green” in many facets in our life, such as reducing the consumption of energy and creating more eco-friendly products as a solution to reduce the crisis that we might face in the future. Computing devices are quickly spreading in our world. Thinking of the amount of devices that are harming the environment through their energy consumption and their toxic wastes urges us to think of a solution that preserves the environment and our well-being. With “quality education” a major issue for the UN 2030 Sustainable Development Agenda, it is a must that we loop together the “quality education” with education per se. It is imperative that we engage the youth in attaining this objective, for it is not enough that we empower them with the tools, we must encourage them to look at the current issues outside the box, through a worldwide view, where the focus nowadays is on the environment, be it in reducing fuel usage, decreasing air and water pollution, and recycling, in brief, being environment friendly. In this paper, we stress the increased number of mobile devices usage and show the importance of having green applications to save the environment and preserve our health. The paper proposes a model with four metrics – energy, economic metric, performance, and energy/performance metric that aims to provide a design for mobile applications that address some of the concerns related to the environment. The proposed model was implemented on a mobile application and the results are compared to a regular application that does not take into consideration the “green” environment. The results proved that the application that follows the defined metrics preserves the environment and performs better than the regular application design. Thus, it presents a stepping stone towards linking learning technology while at the same time sustaining the environment.

## 1 Introduction

Nowadays, we hear the term “Go Green” more and more frequently. We notice flyers all over the streets that encourage us to “Go Green”, and we can see advertisements on the television and on websites that push to “Go Green”. This is because there is a dire need to “Go Green” in order to save the environment and eventually in order to save ourselves. According to statistics that were conducted by the National Academy of Science, there are 14 billion pounds of garbage that are dumped into the ocean every year [1]. Moreover, according to a Pure Earth report, pollution kills 8.9 million people a year and almost 200 million people are exposed to lead, mercury, and pesticides which make them suffer from severe health problems like brain damage [2]. All this is the result of pollution; all these facts pushed the global community to “Go Green”. When it comes to the environment, going green includes practicing procedures that diminish the harm that we are causing to planet Earth. In fact, diminishing the damage people cause to the environment is the only hope that is left to save Earth and save the coming generation. “Going Green” includes all the aspects that can enhance the way we are living in order to protect the environment. However, there is a fact that is not widely known around the world, “Going Green” is not a phrase that is used to emphasize the protection of the environment only. The term is meant to define the concept of renewable, sustainable and eco-friendly procedures, products and energy. Today, there is a great necessity to “Go Green” in many aspects in our life by reducing the consumption of energy and having more eco-friendly products in order to reduce the crisis that we might face in the future.

It is important to “Go Green” in the products that we are using in order to save energy. One of the products that is being widely used all over the world, and is becoming one of the essentials for any person is the computing device. According to the statistics that were conducted by Gartner Inc. in 2013, 2.4 billion computing devices were produced in 2013, which is a 9% increase from the year 2012 [3]. In fact, the production of computing devices is expected to grow reaching more than 2.9 billion devices in 2017 [4]. All these facts allow us to understand the importance of “Go Green” when it comes to computing devices. The term “Green Computing” stands for the process of “Go Green” with computing devices. More precisely, green computing is defined as environmental responsible use of computing devices and all their resources. Almost everyone holds a mobile phone. On average a person uses his/her mobile phone for three hours and sixteen minutes per day [5]. The advantages of the mobile phone tempt people to get one in order to ease their life. Perhaps mobiles are the most common denominator between all people from a socio-economic point of view. It is more commonly used by people from all walks of life irrespective of their socio-economic status. So Go Green is not restricted to only one group of people or one area on planet Earth. The duty is to save the environment of all harm. To reach all people is only possible when we have tailor made campaigns that address each group based on their background (education, wealth, position, etc.). As the development of mobile applications is growing more and more, the mobile phone is not limited to phone calls anymore; there are almost a hundred other features that make the smartphone a significant tool. In this respect perhaps the extra applications to the smart phones are an added value to the less educated and less sophisticated people. Nonetheless, this group still owns a smart phone. According to the journal of Emergency Medicine, a study showed that out of 100,000 patients, 137 more lives were saved when emergency services were called from a mobile phone in case of accidents compared to calling from a landline phone [6]. Here is when the use of a mobile is not restricted to any group.

Over the past ten to fifteen years, community advocacy groups have grown, among others, increasingly concerned with the environmental implications that impact the needs of future generations. Bearing this in mind, it becomes empirical for educational institutions to start at an early stage to investigate the importance of saving the environment. The educational curriculum could include such programs as awareness campaigns under the umbrella of citizenship, civic duty or morals. With technology advancing at such a fast pace, it should be coupled with information on the advantages and disadvantages that it could introduce into our lives. Educators should focus on the importance of not only being technically oriented, but emphasize that being top notch could encompass the values of being a responsible person. The youth should be taught to care about themselves, their families, their neighbors, their community and their country. We should instill in them that present environmental problems would be solved through – the future generations – them, the students; encouraging them to find solutions to the problem of the environment. We should engage them in educational activities (of course after making sure that the students are well-informed about the impact of technology on the environment). Our educational curriculum should be divided into stages, just like any other academic course, each age group with a specific learning outcome. It involves developing the combination of knowledge, skills, values and motivation to make the difference. These activities enrich their lives.

Perhaps, unintentionally technology has paved the path earlier in terms of preserving the trees by introducing the “paperless” office. It is true that we have saved a lot of trees, but this was a by-product of technology – not the core. The more advanced we have become technologically, the more space and fields we can invest in that would lead to a sustainable environment. We are now aware of the dangers that humanity is facing environmentally; it is our duty to safeguard and protect it. We possess the tools. What is needed is the blueprint of twining technology and environment.

Our aim is to show that it is feasible to be innovative technologically and at the same time be environmentally aware of the gains that will ensure as a consequence. Nowadays, more than ever, the “Go Green” slogan has been highlighted and emphasized because of the foreseen dangers that can result if not enough effort is exerted to curb them. As educators, one of the teaching outcomes we should engrave in the minds of the youth is how to couple the education they are getting with innovative methods of serving the environment.

In this work, we propose to “Go Green” in mobile computing. This will be achieved by defining metrics for a green mobile application. In the suggested model, we explore how to deploy a green mobile application that consumes less power, is economic, has a good performance, and decreases the utilization of the device component. A decrease in the utilization will help in protecting the CPU, RAMs, and all of the mobile device's hardware; thus, there will be a decrease in the number of toxic wastes since the mobile device will have a longer lifetime. Our defined green mobile application will not only benefit the environment and protect it, but also it allows the consumers to have a better more satisfied experience with their mobile phones. A green mobile application will save the battery of the mobile phone while enabling the user to benefit from the great features and good performance. In summary, the important contributions of this work are as follows:

• Provide awareness to the youth on the importance of green computing.
• Defining metrics to analyze an application's behavior and decide whether it is green or not.
• Propose a calculation method for each of these metrics that can be adjusted according to the user's preference.
• Implementing a mobile application that follows the proposed metric in order to show that designing a green application is better than a regular one.

The remainder of the paper is organized as follows. Section 2 presents related works. Section 3 announces the data and method. Section 4 presents the results and ensues with a discussion, and section 5 concludes the paper.

## 2 Related work

Green is the term of utmost concerns in our days and was a subject mainly related to environment friendly metrics which has now expanded to computing. Chaudhary et al. [7] discussed the migration from paper to databases in the last decade, and wanted to show that the large storage devices used for saving the databases are posing a threat to the environment as a lot of power is needed to maintain the data centers up and running. Wang and Khan [8] provided a study about the metrics that can help in building a green data center. Blackburn [9] tackled green data centers and presented metrics related to data center's efficiency. Belady [10] discussed data centers power efficiency metrics. In the paper, metrics for measuring power consumption were presented to calculate (specifically) energy consumption and data center's efficiency. Kipp et al. [11] targeted developing methodologies and models to reduce environmental impacts of IT systems. The work focused on the evaluation of IT infrastructure based on the energy efficiency of the hardware environment. Talebi and Thomas [12] presented specific techniques that enable green computing to be integrated in classrooms and research laboratories. These include turning off the equipment between classes, use the computers' power saving modes, eliminate phantom loads, upgrade to extend computer lifecycle, and many other techniques. On a similar note, Mahmoud and Ahmad [13] also discussed techniques and models to build a green application. The paper focused on energy and resources consumptions.

The GREENSOFT model is a well-known model when talking about green software engineering. Naumann et al. [14, 15] attempted to classify green software engineering. The authors explored a software life cycle, and the effects it goes through its different phases. Kocak et al. [16] evaluated the software product and the environmental characteristics of the software using a framework based on ANP, which is a widely used approach to evaluate and prioritize environmental criteria. The main feature in this paper is the developed framework which can help in analyzing and determining the need for each criterion when developing the software model and defining each requirement of the software. Hindle [17] tackled the impact of software evolution and its effect on power consumption by providing metrics to measure power consumption, and by studying the application behavior when it gets updated.

Archiving requires significant storage and process capabilities. In their paper, Bigazzi and Bertini [18] discussed the environmental impact of data archiving and how to add a green measure to it. The paper discussed the sustainability measures in systems, which can be broken into three areas: environmental, economic and social. Atrey et al. [19] discussed the negative aspect that cloud computing has on the environment. In fact, cloud computing provides “unlimited” capabilities; however, the cost for such performance and storage will yield pollution due to the high power consumption and the CO_2_ emission. The work focused on describing the metrics to analyze power consumption. Rivoire [20] presented a green computing model, green metrics and statistics about the adaptability of green computing models. Siso et al. [21] described the negative effects of power and heat consumption in data centers; in addition, they provided metrics to calculate them. Metrics are classified into categories and are ordered by importance.

In our paper, and in contrast with the above discussed papers, we introduce green metrics for mobile applications that will account for the device's entire component in order to reach an optimal green model that will be environment and mobile friendly. In particular, we present metrics that take into consideration the CPU, the memory of the device, the battery and power consumption; whereas, in the previous works, researchers focused on one aspect of a green model, and in particular most of the studies tackle the energy consumption metric and neglect the rest of the components present on the device that might and will harm the environment when neglected during the software design and development process.

## 3 Methods

Developing a well-organized application is always the priority of software engineering companies. Whether it is a mobile application or a desktop application, the aim is always the same – meeting the customers' expectation. However, developing an application requires a good organization where the developers' priorities cannot be limited to the features that the application offers only. In fact, in order to have a fruitful application, several aspects should be taken into consideration including:

- the energy consumption of the application,
- the economic aspect,
- the performance of the application, and
- the utilization of the device components.

Also realizing that the end receivers are not only the educated and business people but people from all walks of life, our proposed model takes into consideration the importance of preparing a well-informed and enlightened end user. Thus, we should prepare basic awareness campaigns starting from the “ABCs” of the concept of Go Green to the place where we prepare to take it.

### 3.1 Energy metric

Developers, nowadays, face the challenge of optimizing the power consumption of their applications; even though, it can be an easy task to perform once the subject is studied in a thorough manner. According to Wainainah [22], mobile phone holders in Kenya, for instance, expect that their phones' battery will last at least eight hours. In fact, if the battery of the mobile phone lasts less than the expected time, many consumers' work might and will be affected. The battery of the mobile phone gets drained as the applications consume a lot of its energy. In fact, the applications that are consuming most of the mobile's battery power are explicitly tending to hurt the environment; because the consumers tend to update their mobile phone with new and faster features, which will increase the toxic waste that is dumped into the environment. Therefore, in order to protect the environment and satisfy the consumers' needs, it is important to have both hardware and software that are power efficient.

### 3.2 Economic metric

Software development is a challenging and expensive process that requires significant resources and finances. A critical objective for its success is to prove the viability and importance of such software development, in order to attract a sufficient number of potential investors that will finance the project. When developing any mobile application or any software in general, the quality, the reliability and the availability of the software are the basic or essential features for attracting investors. Before financing any project, potential investors evaluate the value of such an investment by comparing the cost with its expected return after analyzing its financial studies. That is why, focusing on the return on investment and the time value of money, is actually essential to increase the interest of targeted stakeholders. The term “Return on Investment or (ROI)” according to the Investopedia [23] is a “performance measure that is used to evaluate the effectiveness of an investment or to compare the efficiency of a number of different investments.” To calculate ROI, the benefit (return) of an investment is divided by the cost of the investment (location rental, software licenses, developer salaries, and any cost that can arise for the project); the result is expressed as a percentage or a ratio as per the following formula: $\text{ROI}\left(\%\right)=\;\frac{\text{Gains}\;\text{from}\;\;\text{Investment}\;\left(\text{\$}\right)\;-\text{Project}\;\text{Cost}\left(\text{\$}\right)\;}{\text{Project}\;\text{Cost}\;\left(\text{\$}\right)}\;\times \;100$. In the formula “Gains from Investment” refers to the profits acquired after selling the investment of interest, which in this case is the green mobile application. Return on Investment is a common financial approach because of its adaptability and easiness. Investors should accept positive ROI projects, since gains from investment will be greater than initial project cost. However, projects that have negative ROI are not attractive for investors because they generate extra cost. On the other hand, the Time Value of Money (TVM) is the idea that money available now, is worth more than the same amount in the future due to its potential earning capacity when properly invested, or deposited in a bank account and generating interest. The sooner the money is received, the more its value will be when correctly invested. The Time Value of Money, using the compound interest, is calculated based on the following formula $\;\text{FV}\left(\text{\$}\right)=\text{Current}\;\text{Value}\left(\text{\$}\right)\;\times {\left(1+\text{Interest}\;\text{Rate}\right)}^{\text{Number}\;\text{of}\;\text{Years}}$. The compound formula always generates a higher amount than the simple interest calculation due to the fact that when compounding, each year's earned interest is added to the original amount; and thus, increases that amount against which interest is calculated in subsequent years.

### 3.3 Performance metric

The main aspect that determines the failure or the success of a mobile application is the performance of the application when it is running on the mobile device. The performance of an application reaches the end users directly and affects their opinion; where they will either be encouraged to use the application or they will simply prefer to uninstall if it does not meet their expectations. Performance is based on the relationship between the quality of service and utilization. Utilization is defined by the CPU, RAM, I/O, and storage usage of the application when it runs, and has a direct impact on the performance, where the higher the utilization, the worse the performance. Thus, the quality of the service of an application is a qualitative measure that specifies the performance of the application, in which, high quality of service implies a high performance. Performance is a ratio that is affected by the quality of service and the utilization.

#### 3.3.1 Quality of service

Nowadays, all users have a high expectation of the applications that they download from the stores on any computing device and specifically on the mobile device, for they and are becoming essential parts of our daily lives whether it is a mobile banking application or a social network application. Consumers will never accept a lagging application that is not smooth when running and that does not have an acceptable – if not an excellent – graphical user interface. In fact, experiencing a more advanced application is allowing users to have a high expectation from any downloaded application, starting from its power consumption, to its performance and quality of service. Moreover, the mobile applications stores value the consumers' opinion in any application and open the opportunity for them to leave their feedback which will even encourage other users to download and buy this application or discourage them. Thus, consumers can have a direct effect on the profit of the application and an application that pleases the consumer in terms of the performance can overcome an application that provides sophisticated features yet have a power performance. Part of the quality of service should address the needs of the common user. Educating the common user is important. Simple, easy comprehensible instructions and advices should be part of the quality of service. If this “education” starts as part of the curriculum in schools for students who are at an early stage have become mobile users, but they are yet not really fully aware of the true meaning and impact of Go Green.

#### 3.3.2 Utilization metric

The utilization metric can be calculated in a quantitative manner. We will present a formula for each metric in this section, and depending on the user or the company's needs, each metric can be given a weight according to its importance.

##### 3.3.2.1 CPU usage

One of the important usages when discussing mobile applications' utilization is the CPU usage. The CPU is mainly the most important hardware component on the device. If the CPU processing limit is reached, the device will stop functioning, have heating issues, and might even break down. Effective use of the CPU will lead to a better and faster running application. Most of the time, mobile devices use a small fraction of their CPU power, and mainly make use of less than 5% of their CPU. The CPU metric will thus be discussed and analyzed in terms of percentage, i.e., the average percentage used by an application while running.

##### 3.3.2.2 RAM usage

A RAM device, allows data to be read and written in approximately the same amount of time regardless of the order in which data items are accessed. As the computer runs programs and works with data, it uses RAM to store the program information. Having a bigger RAM allows the computer to run more complex programs in a faster fashion. RAM also allows users to “multitask,” or work among several open programs. Thus, RAM must be given high priority when designing an application.

##### 3.3.2.3 Usage

A computing device and more specifically a mobile device is highly dependent on I/O operations. In our study, I/O will be measured in unit of time which represents the time needed to perform any I/O operation. In fact, many operations and lots of information must be gathered from the Internet, from the device's external storage, and from the device's external memory card since device storage is limited. Thus, having an efficient I/O operation will lead to a fast software application and a fast device; whereas, long and slow I/O operations will lead to a lagging device and a slow application with bad quality of service. Again, the youth are not interested in all the technicalities. Their main concern is the speed and the availability of the Internet. So, we must inform them of the possible drawbacks.

##### 3.3.2.4 Storage usage

Storage usage is a very vital metric especially when discussing mobile devices and in particular mobile phones since storage on mobile devices is very limited. In fact, several manufacturers have commenced building devices whose storage cannot be expandable through a memory card which might be an issue on the long run since application nowadays are growing bigger and are consuming more storage. In our work, we calculate the amount of storage the application is using in terms of megabytes (MB), from the total storage available on the device and present it in the form of a percentage, to show relatively the importance of the storage used by the application.

### 3.4 Energy/performance metric

The developer must balance between energy and performance in order to have a green application that is first, smooth and runs without lagging on the device; and second, that saves the device's battery without affecting the performance. Usually, current software engineers focus mainly on improving an application's performance without taking into consideration the battery consumption which leads to a very good application with enhanced features and functionalities that provide a good quality of service; however, the application consumes battery in an unbalanced manner which will lead to uninstalling the application as no one wants a dead mobile device after a few hours of use. In order to illustrate the importance of this ratio we will explain it by an example. Consider two mobile banking applications having the same features. Both applications provide the customer with a brief summary of the accounts and credit cards, provide detailed information for each of these entities, allow a transfer to be performed and requesting several banking services online. The differences between these two applications are the following: application A is faster than application B. In fact, application A loads in less than one second and is very fast while switching between screens does not show any sign of lagging; whereas, application B provides a smooth quality of service, but takes around three seconds to load and it takes more time than application A to display information. Nevertheless, application A consumes one percent of the battery every five minutes; whereas, application B uses one percent of the battery every fifteen minutes [24]. Since both applications have the same functionality, the consumer will be more tempted to use application B instead of application A; even though, application A is faster and has better performance. This highlights the need for balancing between both energy consumption and application performance.

### 3.5 Methodology design

After defining the metrics and the steps toward a green mobile application, we will now analyze a developed mobile application that follows the steps of green mobile application. The developed mobile application is an iOS application that is designed for banks. The application is compared with a regular bank application that provides exactly the same features in order to show whether the defined metrics are accurate and to show that a green mobile application can outperform a regular mobile application.

Two builds for the mobile banking application were developed; one version, which is a regular application, that does not take green metrics into consideration. Whereas, the other one, the green version, follows the green metrics defined earlier in order to protect the environment. Both builds of the application have the same features. However, the features are implemented using different algorithms in the two builds. Both applications provide the following features: general information related to the bank, account and credit card information, full transaction history, transfers between accounts (under the mobile banking section), and additional services such as requesting a cheque book, a loan, or any service that the bank has to offer (under products) in an easy and well-organized manner to help the customer perform these operations from any place and at any time.

The main aim of the application is to provide ease of use of the bank's services to the customer, which requires a good quality of service from the application. In addition, the application aims to be always up and running in order to better serve the customer. To confer the application, we will start by a splash screen that opens with the bank logo once the application is requested to be loaded for the first time. Then, a welcome screen with a slider displaying marketing banners from the bank appears and under that slider we can find six buttons that direct the user to a page in the application.

The first button introduces general information related to the bank, the second one describes the bank products, the third button is a plan for the youth, the fourth button directs the user to the map to find the nearest branch, the fifth directs the user to the nearest ATM, and finally the last button directs the user to the online banking page where s/he can find all needed information related to his/her accounts.

As mentioned earlier, two builds for this application exist, which means that the same features have been designed in two different ways in order to model the green metrics and the regular software engineering that is currently popular among developers. First of all, one build has been named “greenapp”, which represents our green model while the other build named “redapp”, which is the regular model. Each one of these builds makes use of the device resources in different ways; while both deliver at the end the same result.

### 3.6 Data collection

We pilot tested the performance of applications with participants and sought their feedback using a quick survey. The participants interacted for thirty minutes with the applications. In order to evaluate the users' feedback of the applications, the authors created a quick survey of five questions in English that compares both applications. Participants completed the survey by answering “Yes” or “No” to the five questions (see Tables 1 and 2).

**Table 1:** List of questions.

1) Would you recommend the application to a friend?
2) Could you notice any battery drainage?
3) Were you satisfied with the application's performance?
4) Would you pay for the service and keep the application?
5) Did the quality of service of the product meet your expectations?

**Table 2:** User feedback.

Questions	Greenapp (N = 50)	Redapp (N = 50)
1) Would you recommend the application to a friend?	45 (90%)	15 (30%)
2) Could you notice any battery drainage?	15 (30%)	45 (90%)
3) Were you satisfied with the application's performance?	45 (90%)	25 (50%)
4) Would you pay for the service and keep the application?	40 (80%)	33 (66%)
5) Did the quality of service of the product meet your expectations?	45 (90%)	10 (20%)

### 3.7 Sample

A convenience sample of 50 young adults was approached from the authors' social network and by snowballing. Participants were Lebanese, aged between 18-35 years, have a university degree, and are technology savvy. All participants could speak, read and write Arabic and English.

## 4 Results and discussion

The data in the sample was analyzed using SPSS version 23. Descriptive statistics were computed for the demographic characteristics of the participants as well as the results of the Survey. The sample consisted of equal distribution of genders. Simple descriptive analyses revealed promising performance of the greenapp as opposed to the redapp whereby the majority of participants were satisfied with the greenapp in terms of battery consumption as well as quality and generally met their expectations (see Table 2). In the following sections we discuss the energy, economic, performance, and utilization results.

### 4.1 Energy experiments

In this section, we start by discussing the energy improvement that the green application brings to the device's battery and the impact this enhancement will lead to.

#### 4.1.1 Battery consumption experiment

In order to preserve the battery, first the device's hardware must be battery efficient. Second, the software and applications installed on this device must be battery friendly in order to save as much energy as possible. In our designed application, each build uses the battery of the application in a different way. In fact, “redapp” makes use of the CPU, the RAMs, the mobile network, the Wi-Fi adapter and the GPS as much as possible; whereas, the “greenapp” only uses the CPU, RAM and mobile network to perform the needed tasks. As shown in Table 3, the “redapp” consumes more than double the battery consumption of the “greenapp”. The values of the battery consumption are based on the work of Perrucci et al. [25] – a 2% CPU usage consumes 55 mW/hour, which gives us for the 1% usage a consumption of 27.5 mW/hour. As for the RAM usage, we measured both applications RAM usage and found that “redapp” uses 1.5 times more RAM then “greenapp”, which implies an increase of battery consumption by 1.5. Perrucci el al. also measured the mobile network consumption of battery, and according to their paper, downloading at a rate of 44 kb/sec will induce a usage of 500 mW/hour. According to the iOS system, our application downloads on average 758 kb for the first run and in case of any update at the level of the application the new content will be downloaded incrementally; which implies that with a data speed of 44 kb/sec it will take $\frac{758\left(kb\right)}{44\left(kb/sec\right)}=17\;\text{seconds}$ to download the needed content; thus, the “greenapp” will consume 2.36 mW/hour to download the needed data and store it on the device cache. However, “redapp”, will download the same needed content on every run that is executed. For this reason, if the application is launched twice every hour, it will consume 4.72 mW/hour. The same calculation will be performed for the Wi-Fi adapter, which is used by the “redapp” only; however, the Wi-Fi technology downloads at an average speed of 700 kb/s and consumes 1629 mW/hour. Thus, the application will consume on each run 0.45 mW/hour. Finally, regarding the GPS power consumption which is only used by the “redapp”, when the GPS is operated it consumes 429 mW/hour. If the application is used for around 5 minutes, which is the average usage time for a banking application, we get a battery consumption of 35.75 mW/hour. Table 3 summarizes the battery consumption results.

**Table 3:** Battery consumption.

Resources (mW/hour)	Greenapp	Redapp
CPU	27.5	27.5
RAM	10.2	15.6
Mobile network	2.36	4.72
Wi-Fi	0	0.45
GPS	0	35.75
Total	40.06	84.02

This reduction in component usage affects the energy consumption drastically and improves the lifetime of the battery life.

### 4.2 Economic experiments

Going green provides the investors with a paradigm that offers economic growth while protecting Earth and its' inhabitants. Investors seek to gain profits in any project and green metrics can attract them and satisfy their interest in many ways. It is rather difficult to discuss economics when talking about a computer application as it is not a fixed investment. In order to assess whether the “greenapp” is economically green, we need to specify the cost of the application. Table 4 lists approximate costs of developing the “redapp” and “greenapp” applications. As shown in the table, the cost of the “greenapp” is more than the regular one. The cost of the platform, application design, quality assurance testing, and the publication of both applications is the same since the requirements of both applications are the same. The “greenapp” does not require any extra processor or database space.

**Table 4:** Project cost.

Project cost ($)		Greenapp	Redapp
Fixed costs	Platform cost	5,000	5,000
	Application Design	2,000	2,000
	Submit to iStore	99	99
Variable costs	Development	4,000	3,000
	QA Testing (backend and usability testing)	2,000	2,000
	Total	13,099	12,099

Although the cost of the “greenapp” is higher than the “redapp” application, the “greenapp” application has a higher gain of investment. The “greenapp” application is light on the device as it makes use of fewer resources than the “redapp”. This encourages consumers to use the application. Both applications were tested on a group of fifty young adults to take into consideration the feedback of the consumers. The users were asked the questions listed in Table 5 to test the usefulness of the “greenapp” application.

**Table 5:** User feedback.

Questions	Greenapp	Redapp
1) Would you recommend the application to a friend?	- Yes 45/50 - No 5/50	- Yes 15/50 - No 35/50
2) Could you notice any battery drainage?	- Yes 15/50 - No 35/50	- Yes 45/50 - No 5/50
3) Were you satisfied with the application's performance?	- Yes 45/50 - No 5/50	- Yes 25/50 - No 25/50
4) Would you pay for the service and keep the application?	- Yes 40/50 - No 10/50	- Yes 33/50 - No 17/50
5) Did the quality of service of the product meet your expectations?	- Yes 45/50 - No 5/50	- Yes 10/50 - No 40/50

As per Table 5, users preferred the “greenapp” application and would keep on paying for using the services provided by this application. However, the number of satisfied users drops to almost half when talking about the “redapp”. Suppose that the bank is charging the customers $2 per month for this service. Based on question 4, the bank will earn $960 per year in offering the “greenapp” service; in contrast, the bank will earn only $792 per year in case of providing “redapp” services based on the considered population. This shows that the gain of investment of the “redapp” is almost two third the gains of “greenapp”. On average, a bank has at least 10000 customers and according to our questionnaire, 80% of those customers will subscribe to the mobile banking service which means that the “greenapp” profit will be $16000 every month; whereas, the profit from “redapp” will be $13200 (66% of the customers will subscribe to “redapp”).

In order for potential investors to include the “greenapp” in their investing decisions, ROI of the “greenapp” must be greater than the “redapp”. The ROI of the “greenapp” is $ROI\left(\%\right)=\;\frac{16000\left(\text{\$}\right)\;-13099\left(\$\right)\;}{13099\;\left(\text{\$}\right)}\;\times \;100=\;22.14\%$; in contrast, the ROI for “redapp” will be $ROI\left(\%\right)=\;\frac{13200\left(\text{\$}\right)\;-12099\left(\$\right)\;}{12099\left(\text{\$}\right)}\;\times \;100=\;9.09\%$. This proves that the “greenapp” is a better investment than the “redapp” since it has a greater ROI which will lead to greater return on investment.

Another financial measurement is the payback period, which is calculated in order to know the period that both builds of the application will require to recover the initial expenses of the project. Accordingly, potential investors must seek the project that has a lower payback period for them to recover initial costs earlier. The initial cost of the “redapp” is $12099 and the gain of investment is expected to be $13200 per year as long as the application is active. Thus, the payback period of the “redapp” is 10 months (12099$/13200$=0.91). In contrast, the payback period of the “greenapp” is 9 months only (13099$/16000$=0.81). This allows us to conclude, that the “greenapp” is faster by one month than the “redapp” in recovering the initial cost. The payback reassures results obtained by the ROI method, whereby the “greenapp” is better for investors in comparison with the “redapp”. However, the payback method does not account for time value of money concept and to discount rate. Therefore, the returns generated from both applications, should be compared at the same period taking into consideration the rate of return for each project. To do so, the Future Value of the initial investment should be calculated for both projects for the same investment period. Future Value is the value of an asset or cash at a specified date in the future that is equivalent in value to a specified sum today. For simplicity, three factors in the analysis are assumed: 1) Life term of the projects is equal to 5 years, 2) Initial cost is the Present Value and no other cash flows are considered, and 3) Rate of return is assumed to be 12% for “greenapp” and 10% for “redapp”. The interest rate for the “greenapp” is higher than the “redapp” because the green project is a new project and more risky compared to a regular application that has been in development for years. Risky projects have higher rates of return. The rate of return can be either a cost or an opportunity. However, since the project is an investing project (and not borrowing), then the rate of return will present an opportunity of the initial cost of each project which reflects the present value. Investors must seek projects with greater future value taking the life time of the projects to be the same. Future value is calculated as: FV = PV(1 + R)ˆN whereby PV is the Present Value, R is the discount rate or rate of return, and N is the time. According to the formula, the future value for “redapp” is $FV\left(\$\right)=12099\left(\$\right)\;\times {\left(1+0.1\right)}^{5}$= $19485.56, whereas the future value for “greenapp” is $FV\left(\$\right)=13099\left(\$\right)\;\times {\left(1+0.12\right)}^{5}=\$23084.91$. The FV of 5 years investment in the greenapp is greater by $3599.35, which means that the “greenapp” is a better investment compared to “redapp”.

### 4.3 Performance experiment

The most important metric in our work is the performance, since it will be the main game changer when discussing both builds of the application. In fact, the most important factor in any application for most consumers, developers, and students is the performance and the quality of service of an application. The richer an application is with features, the more its performance gets affected in case it was neglected.

#### 4.3.1 Quality of service

Most users are not looking for complicated applications that require scores of knowledge to use, but prefer simple applications that have a good design and the basic features the application should offer [26]. Here this applies to all consumers, be it an investor, a student, or an ordinary grocery shop owner. Thus, one can say that having extra features on an application is not always a good idea. Based on our questionnaire in Table 5, we can conclude that 45 out of the 50 testers were satisfied with the quality of service provided by the “greenapp” application, in contrast to only 10 out of the 50 who appreciated the quality of service of the “redapp”. This shows that the quality of service of the “greenapp” is better than the “redapp” since it has an enhanced performance.

#### 4.3.2 Utilization experiment

Each feature implemented in the mobile application will require some component resources to work properly; and thus, will affect the overall performance of the application. In our case, we made use of several green techniques while developing “greenapp” in order to have our green application.

##### 4.3.2.1 CPU and RAM experiment

First of all, let us discuss the performance differences of both applications in terms of implementation. Also, keeping in mind not only the well-informed but also those who are ordinary users and not knowledgeable enough with the terms we are using. The application allows the user to get the location of the nearest branch and the nearest ATM available. In order to do so, the current location of the user must be fetched by the application to compare it with the available branches or ATMs to return to the requester a list of the nearby branches (or ATMs). In order to provide the service, the developer can implement this feature in two different ways. The first by using the GPS system built into the device and the second by using the mobile network. Both solutions will lead to the same result; however, using the GPS will incur additional power since a new component of the computing device is used (in addition to using more CPU power and more RAM space). Conversely, when using the mobile network to get the user's location, the CPU usage will not be affected, RAM storage will not increase, and the power will not be affected since any regular mobile phone user is always connected to his/her cellular network in order to receive calls. Thus, this mobile component is always in use by the device. We can now say that, both applications will provide a list of the nearest branches/ATMs. The location service will use more memory when the GPS component is used instead of the mobile network. The application “greenapp” memory consumption is 93 MB, while “redapp” consumes 130 MB from the random access memory.

Another important feature that was implemented in our study is the concept of garbage collection and tasks killing. Once the application is launched and loaded, it will use RAM space and consume data usage (Internet consumption) when it needs to fetch data from the Internet. The most important concept in this case is resources allocation. Once the application is hidden in the background (user switches from one application to another), it should either become idle or get killed. In our case, we decided to put the application to sleep. In other words, once the application is in the background, it will release a part of the memory and will have an active listener that will wake the application from its idle state when the user switches back to it. This will lead to a better performance since the application will not be loaded several times and will decrease CPU consumption as it is already present in the random access memory. In our mobile banking application, we use the memory allocation function in order to keep the RAM free as much as possible. When running “redapp”, if the user accesses the map and closes it several times, the RAM usage will keep on increasing indefinitely; this is because the memory is not being freed when the user exits from a screen. In contrast, in the “greenapp” the memory is being freed whenever the user exits from a screen. This enables the mobile device to have higher free RAM space available when there is a task request for using it. The “greenapp” memory is being freed; thus, it uses only extra 11.5 MB from the RAM compared to 29.5 MB in the case of the “redapp” which is more than double the usage. The RAM consumption is mainly stable on “greenapp”, whereas in “redapp” the RAM will keep on increasing until no more space exists on the device and the application will crash.

##### 4.3.2.2 I/O and storage experiment

Another important feature in the “greenapp” build is the use of caching. In fact, when the application is launched, the home screen slider will provide the user with the latest marketing campaigns available from the bank. Thus, this slider needs to be regularly updated with the latest campaigns for consistency. One of the ways to develop this feature, which is done on “redapp”, is by getting all the marketing campaigns from the Internet each time the application is launched and the slider is swiped. This will lead to high data consumption since the application will be mainly downloading pictures. However, it will lead to a bad quality of service when working with the slider and swiping through the campaigns in the case of a slow or bad internet connection. This will incur lagging in the application which will negatively affect the quality of service. Fetching all this data from the internet is a “unscrupulous” option, used by most of the developers since it is the easiest solution; however, it will lead to high data consumption and bad quality of service which are two important factors for any mobile phone user. In order to improve and solve this issue, “greenapp” makes use of caching and storing all this information on the device's storage component, temporarily with the date of the last update (date of last time the cache was updated). Such a design will affect the performance of the application since the “redapp” depends on the internet to fetch all needed data and display them on the slider. This will lead to a higher cost in terms of bandwidth and I/O usage with respect to the “greenapp” usage of I/O and bandwidth. Both applications need to fetch 758 KB from the internet; however, “greenapp” will perform that operation once and store the data on the device's storage; whereas, “redapp” will download that information each time the application is executed which will lead to high I/O usage and lagging in the display of the pictures due to the data transfer that might not be always stable. Although, the “greenapp” saves I/O usage, it will store the data on the device's storage. Hence, the “greenapp” uses more device storage than the “redapp”.

### 4.4 Energy/performance experiment

This metric accounts for balancing between the two separate individual metrics in order to reach, if possible, the optimal green application in terms of both performance and energy consumption. In order to reach such a balance, the developer must take care of both performance and energy issues starting from the design phase. The “redapp” build was developed regularly; with only application features and performance in mind while ignoring the impact this will induce on the battery and the user experience. Table 6 summarizes the results induced and shows that “greenapp” utilization overcomes “redapp” utilization in terms of CPU, RAM, and I/O usages; however, “redapp” has slightly a better utilization in storage usage.

**Table 6:** Performance utilization values.

	Greenapp	Redapp
CPU usage	1%	1%
RAM usage	93.4 MB	130 MB
I/O usage	758 KB	758KB×timesapplicationlaunched
Storage usage	9.658 MB	8.9 MB

The better performance affected the energy consumption of the “greenapp” as shown in Table 6. The usage of the CPU in both applications is the same (1%); thus, the energy consumption of the CPU in both builds is also the same (27.5 mW/hour). However, the RAM usage in the “redapp” is higher than the “greenapp” which impacted the battery consumption where the “redapp” consumes 15.6 mW/hour while the “greenapp” consumes only 10.2 mW/hour. Moreover, the I/O usage of “greenapp” is lower than the usage of “redapp”; this fact imposed less consumption of energy in the “greenapp” application where the mobile network consumed only 2.36 mW/hour while “redapp” 4.72 mW/hour. In order to calculate the Energy/Performance ratio, we sum up the RAM, I/O, and storage usage in MB; the CPU usage is going to be neglected since it is the same between the two applications. Moreover, the battery consumption of all the resources except the CPU is going to be calculated to measure the effect of the performance on the energy (see Table 7).

**Table 7:** Energy/performance values.

Application	Greenapp	Redapp
Energy summation of the resources	12.256 mW/hour	56.52 mW/hour
Performance utilization summation	103.816 MB	140.416 MB

As shown by the figures in Table 7, the performance utilization summation of the “greenapp” is better than the “redapp”, which had an impact on the energy consumption of the resources where the “redapp” consumes 44.264 mW/hour more than the “greenapp”. This being said, we can conclude that the balance between the performance and the energy metrics was successful in the “greenapp” which, overall, is better than the “redapp”.

Through teaching the students about saving Planet Earth, we are helping them see the difference between the centrality of knowledge versus action – we show them the importance of morality and ethics, of being a citizen of a planet they are worth of because they have helped shape this planet, to be better by caring and working. We aim through this work to be sensitive and understanding of the environmental problems as well as addressing it through collaboration and commitment.

As for the commoners, the TV programs, simple advertisement, help-line centers, social gatherings and simple awareness campaigns are stepping stones to reduce the negative impact of technology and increase the Go-Green. Green flyers, green phones, green chairs, green bottles, coloring everything in green could send the message. To do this, arts and cultural organizations must understand themselves not as arbiters of taste, but as creative homes for people. They must be platforms for cultivating public awareness.

## 5 Conclusion

Mobile phones expansion is growing exponentially nowadays. Every person has at least two mobile devices and this growth comes with a negative effect on the environment. In fact, the development of a mobile device requires a lot of machines to be used and many hazardous elements combined together which have a negative effect on the environment. There are several ways to limit such impact on the environment by mainly taking care of developing mobile friendly software that will run on the device, following some predefined procedures which will lead to a green application being deployed which will protect the environment and increase the device lifetime. In our work, we introduced a model based on four metrics: The energy metric tackled battery consumption issues where several solutions were provided in order to decrease the battery draining. The economic metric dealt with the return on investment, the future value of money, and the project's payback. The performance and utilization metric take care of the usage of the device's resources where we defined and presented several formulas to study these usages and provided solutions to reduce them in an efficient manner that does not affect the quality of service. The last metric is a combination of the performance and energy metrics where we provided a way to balance between both applications to get the best result and reach an optimal green and eco-friendly application that has a balanced usage.

Once these metrics were defined we developed an application that follows them in order to demonstrate and prove their effectiveness. The developed application showed positive results and proved that a green application is better than a regular application.

The current research is focused on the effectiveness of active teaching learning strategy to address the environmental issues. This will add to the existing body of literature with important insights regarding how best to develop programs that promote pro-environmental behavior and sensitize the student community about the various complex local environmental issues. When service activity is integrated with academic curriculum and content, students engage in reflection activities that apply their learning in real life activities. This input from educational institutions is a limitless source of a future that has yet to be fully explored. We are living in fear of what is to come and happen to our planet Earth. Without hope, we are devoid of imagination and effectiveness that allows us to lift up and out of our problems to reach for a better tomorrow.

We want end users and particularly the youth to recognize that being environmentally friendly is and must be seed in relation to being technologically advanced. We also want research to focus on the effectiveness of active teaching learning strategies to address the environmental matters. This will add to the existing body of literature with important insights regarding how best to develop “green” programs that promote pro-environmental behavior and sensitize the youth community about the various complex local environmental subjects. They can go hand in hand with neither of them negating or hindering the other's progress. We hope more awareness to the “go Green” is highlighted and the youth get more engaged in exploiting their creative minds in investing their information in this area. After all, the environment does not belong to one particular nation, people, etc. The environment is for all. So keeping it green is for all!!

To sum up, this presented research aims to bridge the gap between advancement in technology and saving the environment; in other words, the relationship between moral foundation endorsement and computing devices. The association between moral foundation endorsement and technology has received more recent attention, yet limited to some areas more than others. The need to highlight a set of basic universal concerns that guide and deal with the protection of the environment. Only through planning the seeds of responsibility in the minds of the youth, and highlighting the “uninformed” user as well as arguing with the educator can we full heartedly say that we have a “Green” future awaiting planet Earth.

Our work is based on pilot testing. An inherit limitation of pilot testing is that the findings cannot be generalized to the overall population of interest. To overcome this limitation, we plan, in the future, on conducting the questionnaire on a bigger sample size that is more representative of the general population using the appropriate test statistics and power analysis.

We also plan to develop each metric more thoroughly with more details to try and reach the optimal green application. In addition, since the computation of the metrics is rigid, a formula will be developed that can cater for all the defined metrics. In other words, this formula will be able to give information on how the application is. It will also have weights for each metric. Thus, in this way the investors will have the ability to express their interest in each metric, and give each metric a different degree of importance according to their need and to the project's need. Moreover, the scope of our research is going to be expanded to cover all the computing devices and will not be limited to smartphones especially that the future predicts an increase in the usage of the computing devices in every aspect of our lives.

## Declarations

### Author contribution statement

Ramzi A. Haraty, Georges Bitar: Conceived and designed the experiments; Performed the experiments; Analyzed and interpreted the data; Contributed reagents, materials, analysis tools or data; Wrote the paper.

### Funding statement

This work was supported by Lebanese American University.

### Competing interest statement

The authors declare no conflict of interest.

### Additional information

No additional information is available for this paper.
